# Predictive biomarkers of response and survival following immunotherapy with a PD-L1 inhibitor benmelstobart (TQB2450) and antiangiogenic therapy with a VEGFR inhibitor anlotinib for pretreated advanced triple negative breast cancer

**DOI:** 10.1038/s41392-023-01672-5

**Published:** 2023-11-17

**Authors:** Yiqun Han, Jiayu Wang, Tao Sun, Quchang Ouyang, Jianwen Li, Jie Yuan, Binghe Xu

**Affiliations:** 1 National Cancer Center/National Clinical Research Center for Cancer/Cancer Hospital, Chinese Academy of Medical Sciences and Peking Union Medical College, Beijing, 100021 China; 2 Liaoning Cancer Hospital & Institute, Shenyang, Liaoning 110042 China; 3 Hunan Cancer Hospital, Changsha, Hunan 410013 China; 4 Geneplus-Shenzhen, Shenzhen, 518118 China

**Keywords:** Prognostic markers, Predictive markers, Breast cancer

## Abstract

In our phase Ib trial (ClinialTrials.gov Identifier: NCT03855358), benmelstobart (TQB2450), a novel humanized IgG1 antibody against PD-L1, plus antiangiogenic multikinase inhibitor, anlotinib, demonstrated promising antitumor activities in pretreated triple negative breast cancer (TNBC) patients. We conducted explorative analyses of genomic biomarkers to explore the associations with treatment response and survival outcomes. Targeted next generation sequencing (NGS) was undertaken toward circulating tumor DNA (ctDNA) collected from peripheral blood samples prior to the start of treatment and after disease progression. A total of 31 patients received targeted NGS and functional driver mutations in 29 patients were analyzed. The most frequent mutations were *TP53* (72%), *MLL3* (28%), and *PIK3CA* (17%). At a blood-based tumor mutational burden (bTMB) cutoff of 6.7 mutations per megabase, patients with low bTMB showed better response to anlotinib plus TQB2450 (50% vs. 7%, *P* = 0.015) and gained greater PFS benefits (7.3 vs. 4.1 months, *P* = 0.012) than those with high bTMB. At a maximum somatic allele frequency (MSAF) cutoff of 10%, a low MSAF indicated a better objective response (43% vs. 20%) as well as a significantly longer median PFS (7.9 vs. 2.7 months, *P* < 0.001). Patients with both low MSAF and low bTMB showed a notably better objective response to anlotinib plus TQB2450 (70% vs. 11%, *P* < 0.001) and a significantly longer median PFS (11.0 vs. 2.9 months, *P* < 0.001) than patients with other scenarios. Our findings support future studes and validation of MSAF and the combined bTMB-MSAF classification as predictive biomarkers of immune checkpoint inhibitor-based regimens in advanced TNBC patients.

## Introduction

Triple negative breast cancer (TNBC), an aggressive subtype of breast cancer, has an extremely unfavorable outcome, with a median overall survival (OS) between 12 and 18 months.^1,2^ Cytotoxic chemotherapy remains the standard therapeutic modality, but treatment response is suboptimal and not durable, and the tumor eventually progresses, relapses, or becomes metastatic in most patients on first-line chemotherapy.^3^ Pretreated TNBC patients have rather limited therapeutic options, with standard chemotherapy having a low response rate (10 to 15%) and a progression-free survival (PFS) of merely 2 to 3 months, highlighting the need for novel therapeutic options for these patients.^4^

TNBC is amenable to immunotherapy because of high levels of PD-L1 on both tumor and immune cells and the presence of tumor infiltrating lymphocytes.^5,6^ However, in pretreated TNBC patients, single agent immune therapy has demonstrated poor efficacy.^7^ In the phase II KEYNOTE-086 study, pembrolizumab achieved an objective response rate (ORR) of 5.3% in pretreated metastatic TNBC patients not selected for PD-L1 status, and there was no improvement in ORR (5.7%) in the PD-L1-positive subpopulation.^8^ Similar observations were made in clinical trials with other PD-L1 inhibitors including avelumab and atezolizumab.^9,10^ In the phase III KEYNOTE-119 study, pembrolizumab did not improve OS, the primary endpoint of the study, and PFS *versus* chemotherapy in pretreated metastatic TNBC patients.^11^ Vascular endothelial growth factor (VEGF), which is expressed in 30–60% of TNBC, drives aberrant angiogenesis and represents a prime target for molecular targeted therapy.^12^ Nevertheless, several trials have shown that the addition of monoclonal anti-VEGF antibody bevacizumab to standard of care for TNBC failed to improve disease-free survival or OS in TNBC.^13–15^

The lack of robust results with either single agent immune therapy or antiangiogenic therapy highlights the need for combination strategies to tackle this challenging disease. Next-generation sequencing (NGS) study has revealed that primary TNBC is a mutationally heterogeneous tumor at the time of diagnosis with wide variations in gene mutational patterns and pathway involvement.^16^ There have been scant investigations on genomic profiling in advanced TNBC (aTNBC). Currently, PD-L1 remains the only validated predictive biomarker of immune therapy for breast cancer.^17^ Hitherto, no predictive biomarker has been identified and validated that would enable molecularly stratified therapy and no effective biomarkers have been established for combined immune therapy and antiangiogenic therapy of aTNBC, which are critical for identifying patients who could benefit from the combination therapy while avoiding the cost and toxicity of such treatments in patients who are unlikely to respond. Development and incorporation of tumor and blood-based biomarkers of treatment response to combined immune therapy and antiangiogenic therapy would allow for a molecularly stratified selection of suitable patients, thus paving the way for more precise and personalized combinational therapy.

Our phase Ib trial (ClinialTrials.gov Identifier: NCT03855358) enrolled patients with histopathologically confirmed pretreated aTNBC. The patients received combined immune therapy and antiangiogenic therapy with benmelstobart (TQB2450), a newly-developed humanized IgG1 antibody against PD-L1 plus escalating doses of anlotinib, a multikinase inhibitor (MKI) that suppresses oncoangiogenesis by simultaneously blocking VEGFR, FGFR, PDGFR, and c-Kit.^18^ The study achieved an ORR, the primary endpoint, of 26.5% (9/33, 95% CI 13.0–44.0), which was reported separately. In the current report, we conducted thorough explorative analyses of the gene mutational profile and, particularly, genomic biomarkers to predict the treatment response and survival outcomes of the study cohort in the phase Ib trial.

## Results

**Fig. 1:**
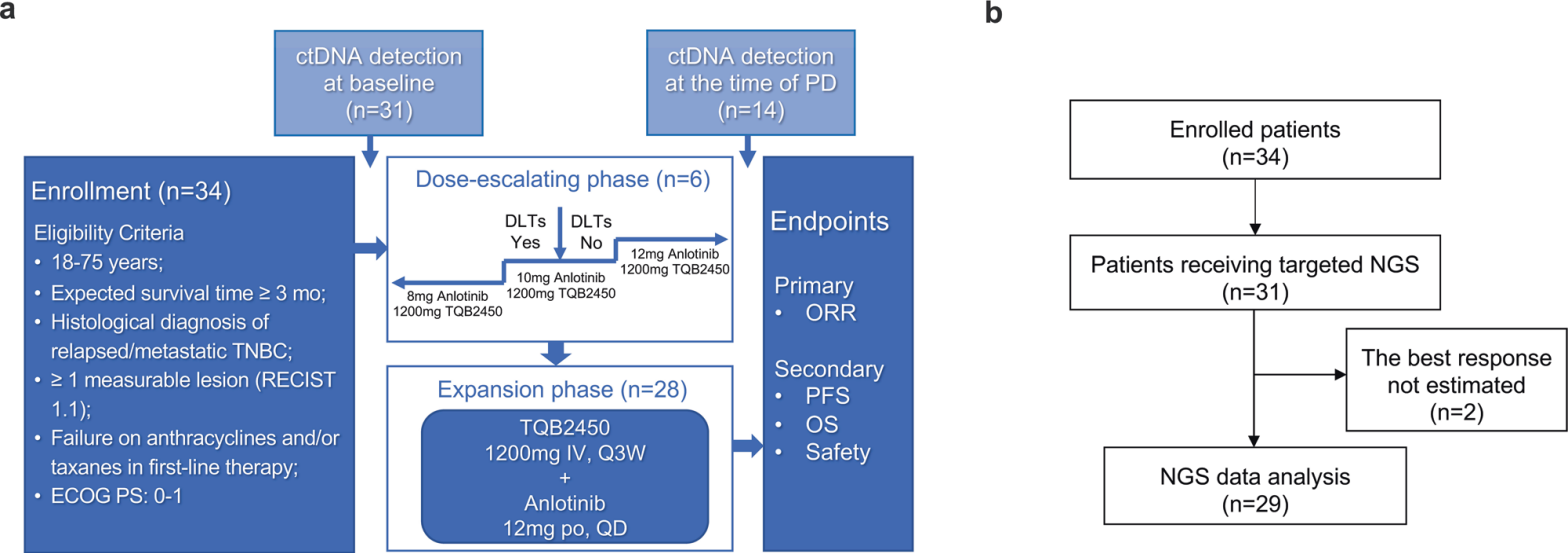
The flow diagram of the study. **a** The flowchart of the clinical trial and NGS. **b** Selectivity and eligibility of patients receiving NGS at baseline. ctDNA, circulating tumor DNA. DLT dose limiting toxicities, NGS next generation sequencing, PD progressive disease, ORR objective response rate, PFS progression-free survival, OS overall survival

Thirty-four aTNBC patients were eligible for the study (Fig. 1a). Totally 31 patients received targeted NGS and functional driver mutations in 29 patients were analyzed after excluding 2 patients whose best overall response was not evaluable (Fig. 1b).

### Genomic landscape of aTNBC

**Fig. 2:**
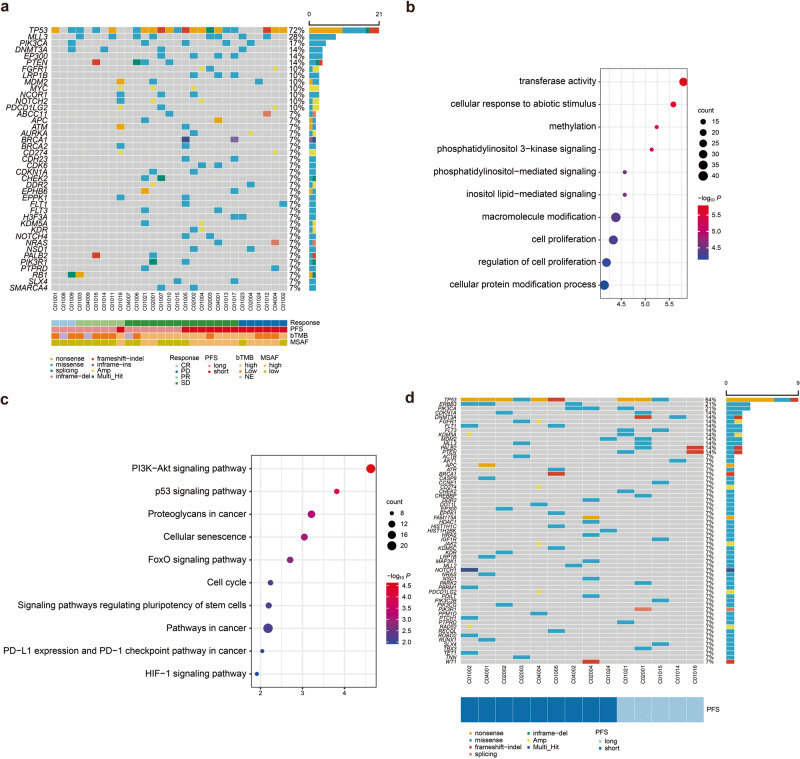
**a** Genomic landscape of relapsed or metastatic triple negative breast cancer. OncoPrint of functional driver mutations in 26 patients with triple-negative breast cancer. Genes altered in at least 7% of the cases are shown. Rows represent genes and columns represent individual samples. Glyphs and color coding are used to summarize distinct genomic alterations including mutations, copy number alterations (amplifications, deletions, and insertions), best response, PFS status, and TMB. PFS long ≥4.9 months; PFS short <4.9 months. TMB high ≥5 Muts/Mb; TMB low <5 Muts/Mb. MSAF high ≥13%; MSAF low <13%. CR complete response, MSAF maximum somatic allele frequency, Muts/Mb mutations per megabase, PD progressive disease, PFS progression-free survival, PR partial response, SD stable disease, TMB tumor mutational burden, WT wildtype. **b** GO enrichment analysis of mutated genes in relapsed or metastatic triple negative breast cancer. Size and color of the bubble represent the number of mutated genes enriched in a pathway, or biological process, and enrichment significance, respectively. **c** KEGG pathway enrichment analysis is summarized by bubble charts. The x-axis shows enrichment factors and the y-axis shows the pathway terms. **d** OncoPrint of functional driver mutations in 14 patients with triple-negative breast cancer after PD

The majority of the patients (90%, 26/29) harbored gene mutations, with a median of 6 mutations (range 0–18). Overall, 96% (25/26) had missense mutations, 46% (12/26) harbored nonsense mutations, and 23% (6/26) carried gene amplification. *TP53* (72%), *MLL3* (28%), and *PIK3CA* (17%) were the three most frequently mutated genes. In addition, *DNMT3A*, *EP300*, and *PTEN* were mutated each in 14% of the patients. *FGFR1*, *LRP1B*, *MDM2*, *MYC*, *NOTCH2*, *PDCD1LG2* and *NCOR* were mutated each in 10% of the patients (Fig. 2a). GO annotation analysis showed that the altered genes were significantly associated with phosphatidylinositol 3−kinase (PI3K) signaling and other cellular processes (Fig. 2b). The mutated genes were further grouped into their respective KEGG pathways with the PI3K/AKT pathway being most enriched in the altered genes, including *PIK3CA* and *PTEN* (Fig. 2c). By contrast, upon disease progression, the 3 most frequently mutated genes were *TP53* (64%), *ERBB3* (21%), and *PIK3CA* (21%). Furthermore, *CDKN1A*, *DNMT3A*, *FGFR1*, *FLT1*, *FLT3*, *KDM5A*, *MDM2*, *MLL3*, and *PALB2* were each mutated in 14% of the patients (Fig. 2d).

### Gene mutations and treatment response in TNBC

**Fig. 3:**
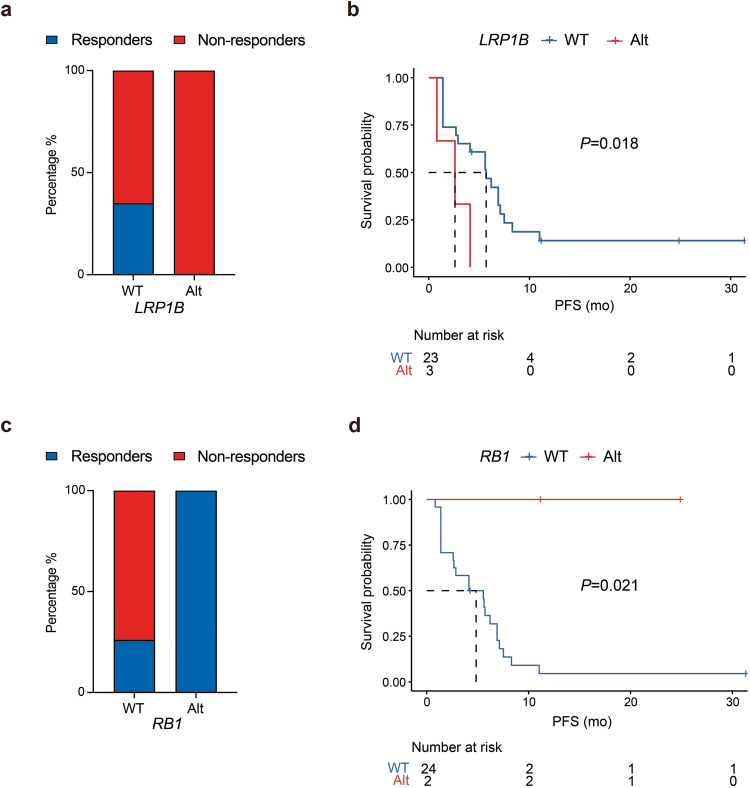
Gene mutations and treatment response in relapsed or metastatic triple negative breast cancer. Treatment response of efficacy-evaluable patients with mutated *versus* wildtype *LRP1B* (**a**) and mutated *versus* wildtype *RB1* (**c**). The Kaplan–Meier curves of progression-free survival (PFS) of efficacy-evaluable patients stratified by the alteration status of *LRP1B* (**b**) and *RB1* (**d**)

We further undertook clinical and genomic analysis to explore potential predictors of response in relapsed or metastatic TNBC. Three patients had mutated *LRP1B*; none of them responded to treatment while 30% (7/23) of the patients who had wildtype *LRP1B* responded to treatment (Fig. 3a). Patients with mutated *LRP1B* had a significantly shorter PFS than patients who had wildtype *LRP1B* (2.8 months *vs*. 6.9 months, *P* = 0.018) (Fig. 3b). In addition, 2 patients had mutated *RB1*, and both responded to treatment, 1 attaining complete response (CR) and the other partial response (PR), while 26% (7/27) of the patients with wildtype *RB1* had a treatment response (Fig. 3c). However, no significant difference was observed in the median PFS of patients with mutated *RB1* and those with wildtype *RB1* (Fig. 3d). Despite a higher treatment response rate in patients with wildtype *TP53 versus* patients with mutated *TP53* (2/5, 40% *vs*. 5/21, 24%), there was no statistically significant difference (Supplementary Fig. 1a). Meanwhile, patients with wildtype *MLL3* had a response rate similar to that of patients with mutated *MLL3* (5/18, 28% *vs*. 2/8, 25%) (Supplementary Fig. 1b). Patients with wildtype *PIK3CA* had a response rate comparable to that of patients with mutated *PIK3CA* (6/21, 29% *vs*.1/4, 25%) (Supplementary Fig. 1c). Patients with wildtype *DNMT3A* had a lower response rate than patients with mutated *DNMT3A* (5/22, 23% *vs*. 2/4, 50%), but there was no statistical difference (Supplementary Fig. 1d). In addition, there was no significant difference in the median PFS of patients with mutated and those with wildtype *TP53*, *MLL3*, *PIK3CA*, and *DNMT3A*.

### Low blood-based TMB (bTMB) is potentially associated with better response to immunotherapy and antiangiogenic therapy in metastatic TNBC patients

**Fig. 4:**
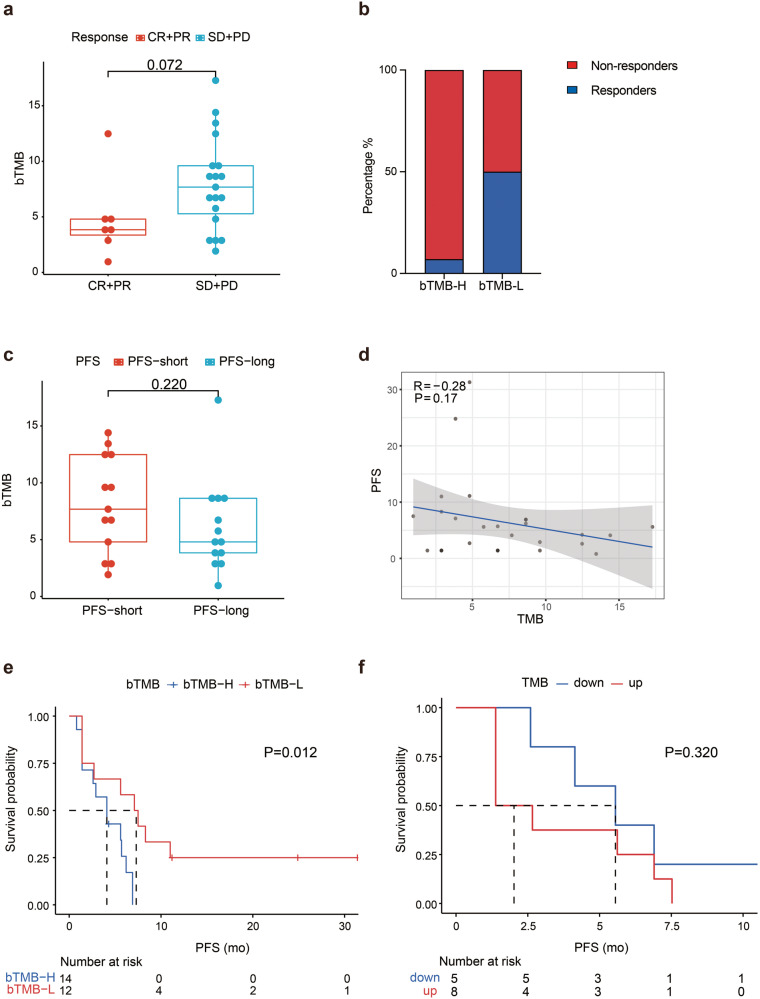
Blood-based tumor mutational burden (bTMB) is associated with clinical outcomes of relapsed or metastatic triple negative breast cancer. **a** Box plots of bTMB in responders (CR and PR) and nonresponders (SD and PD). **b** The percentage of responders and nonresponders in patients with high *vs*. low bTMB. **c** Box plots of bTMB of efficacy-evaluable patients with long *vs*. short PFS. **d** Spearman rank correlation analysis of bTMB and PFS. **e** Kaplan–Meier curves of PFS of efficacy-evaluable patients with relapsed or metastatic TNBC stratified by high *vs*. low bTMB. **f** Kaplan–Meier curves of PFS of efficacy-evaluable patients with relapsed or metastatic TNBC stratified by changes in bTMB post treatment. Median PFS (4.9 months) was used to demarcate long from short PFS and median TMB (6.7 Muts/Mb) was employed to different high TMB from low TMB. PFS long ≥4.9 months; PFS short <4.9 months. TMB high ≥6.7 Muts/Mb; TMB low <6.7 Muts/Mb

Hitherto, no data are available about the relationship between bTMB and responses to combined immunotherapy and antiangiogenic therapy in TNBC. We were interested in whether bTMB could be utilized as a biomarker predictive of treatment responses in relapsed or metastatic TNBC patients. Twenty-nine patients were efficacy evaluable and their clinicopathological characteristics are presented in Supplementary Table 1. Seven patients (7/29, 24%) showed objective response to anlotinib plus TQB2450. Twenty-six patients had data on baseline bTMB, with a median of 6.7 mutations per megabase (Muts/Mb) (range 1.0–17.3). Patients who responded to anlotinib plus TQB2450 tended to have a lower median TMB than those who did not (3.8 Muts/Mb *vs*. 7.7 Muts/Mb), but without apparent significant difference (*P* = 0.072) (Fig. 4a). Furthermore, using median TMB to differentiate patients with high (bTMB-H) from low bTMB (bTMB-L), a greater proportion of patients with low bTMB showed objective response to anlotinib plus TQB2450 than those with high bTMB (50% *vs*. 7%) (Fig. 4b). Patients with short PFS ( < 4.9 months) had a numerically higher median bTMB than those with long PFS (≥4.9 months) (7.68 Muts/Mb *vs*. 4.8 Muts/Mb, *P* = 0.220) (Fig. 4c). Spearman rank correlation analysis demonstrated a weak negative association between PFS and bTMB (r = −0.28, *P* = 0.17) (Fig. 4d). Patients with low bTMB had a significantly longer median PFS than those with high bTMB (7.3 months *vs*. 4.1months, *P* = 0.012) (Fig. 4e). Thirteen patients had plasma DNA data both at baseline and PD; 5 experienced a reduction in bTMB. Patients who experienced a decline in bTMB had a numerically longer median PFS (6.3 *vs*. 4.2 months), but there was no statistical difference (*P* = 0.320) (Fig. 4f).

### Low blood-based maximum somatic allele frequency (MSAF) is associated with better response to immunotherapy and antiangiogenic therapy in relapsed or metastatic TNBC patients

**Fig. 5:**
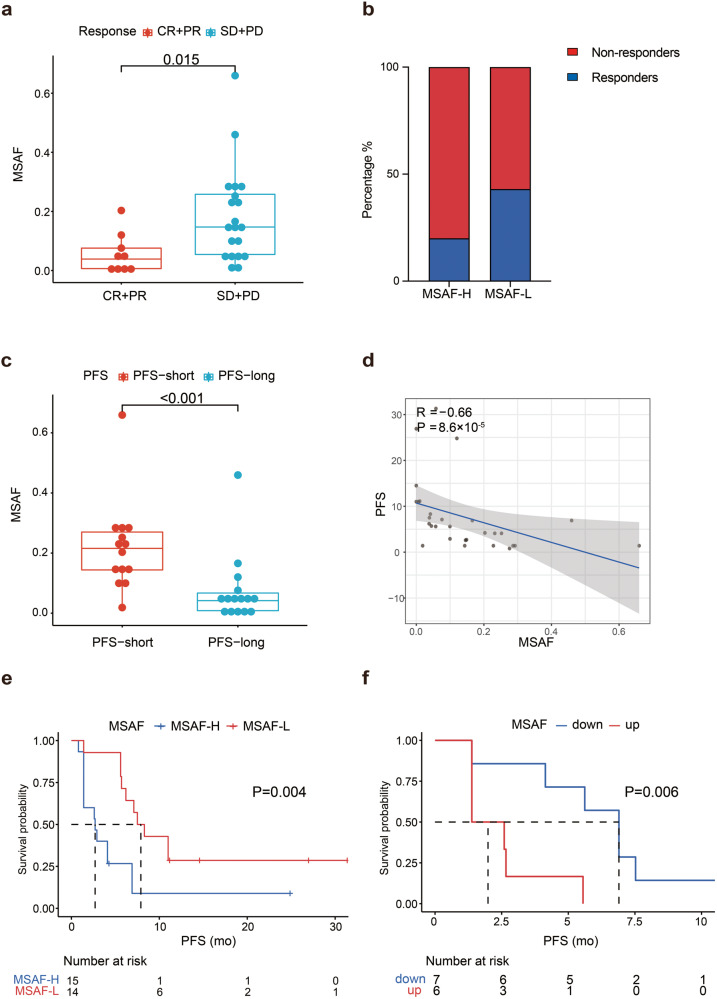
The maximum somatic allele frequency is associated with clinical outcomes of relapsed or metastatic triple negative breast cancer. **a** Box plots of MSAF for efficacy-evaluable patients with relapsed or metastatic TNBC who had treatment response (CR and PR) *vs*. those did not respond to treatment (SD and PD). **b** The percentage of responders and nonresponders in patients with high *vs*. low MSAF. **c** Box plots of MSAF of efficacy-evaluable patients with long *vs*. short PFS. **d** Spearman rank correlation analysis of MSAF and PFS. Cox regression analysis of correlation between MSAF, age, ECOG PS, and visceral metastasis and PFS. **e** Kaplan–Meier curves of PFS of efficacy-evaluable patients with relapsed or metastatic TNBC stratified by high *vs*. low MSAF. **f** Kaplan–Meier curves of PFS of efficacy-evaluable patients with relapsed or metastatic TNBC stratified by changes in MSAF post treatment. Median PFS (5.6 months) was used to demarcate long from short PFS and median MSAF (10%) was employed to different high MSAF from low MSAF. PFS long ≥5.6 months; PFS short <5.6 months. MSAF-high ≥10%; MSAF-low <10%

The concordance between bTMB and tissue-based TMB could be affected by the proportion of tumor-derived plasma DNA and recent studies have shown that bTMB is suboptimal in predicting treatment response in cancer patients.^19^ We investigated whether MSAF, which estimates the fraction of tumor fraction in circulating DNA, could be a potential predictor of responses to combined immunotherapy and antiangiogenic therapy in relapsed or metastatic TNBC patients. MSAF was available in 29 patients, with a median of 10% (0%–66%) and their clinicopathological data are shown in Supplementary Table 2. The responders to anlotinib plus TQB2450 had a significantly lower median MSAF than the non-responders (5.7% *vs*. 14.6%, *P* = 0.015) (Fig. 5a). The median MSAF (10%) was used to separate patients with high from low MSAF. A greater proportion of patients with low MSAF responded to anlotinib plus TQB2450 than those with high MSAF (43% *vs*. 20%) (Fig. 5b). Furthermore, patients with short PFS had a significantly higher median MSAF (21.6%) than those with long PFS (4.2%, *P* < 0.001) (Fig. 5c). Spearman rank correlation analysis demonstrated a moderate and yet significant correlation between MSAF and PFS (r = −0.66; *P* = 8.6 × 10^–5^) (Fig. 5d). The median PFS of patients with a low MSAF was significantly longer than that of patients with a high MSAF (7.9 *vs*. 2.7 months, *P* = 0.004) (Fig. 5e). MSAF was available in 13 patients both at baseline and at the time of progressive disease (PD); MSAF decreased in 7 patients and increased in 6 patients post treatment. The median PFS of patients with decreased MSAF was significantly longer than that of patients with increased MSAF (6.9 *vs*. 2.0 months, *P* = 0.006) (Fig. 5f).

### Correlation of MSAF and bTMB

**Fig. 6:**
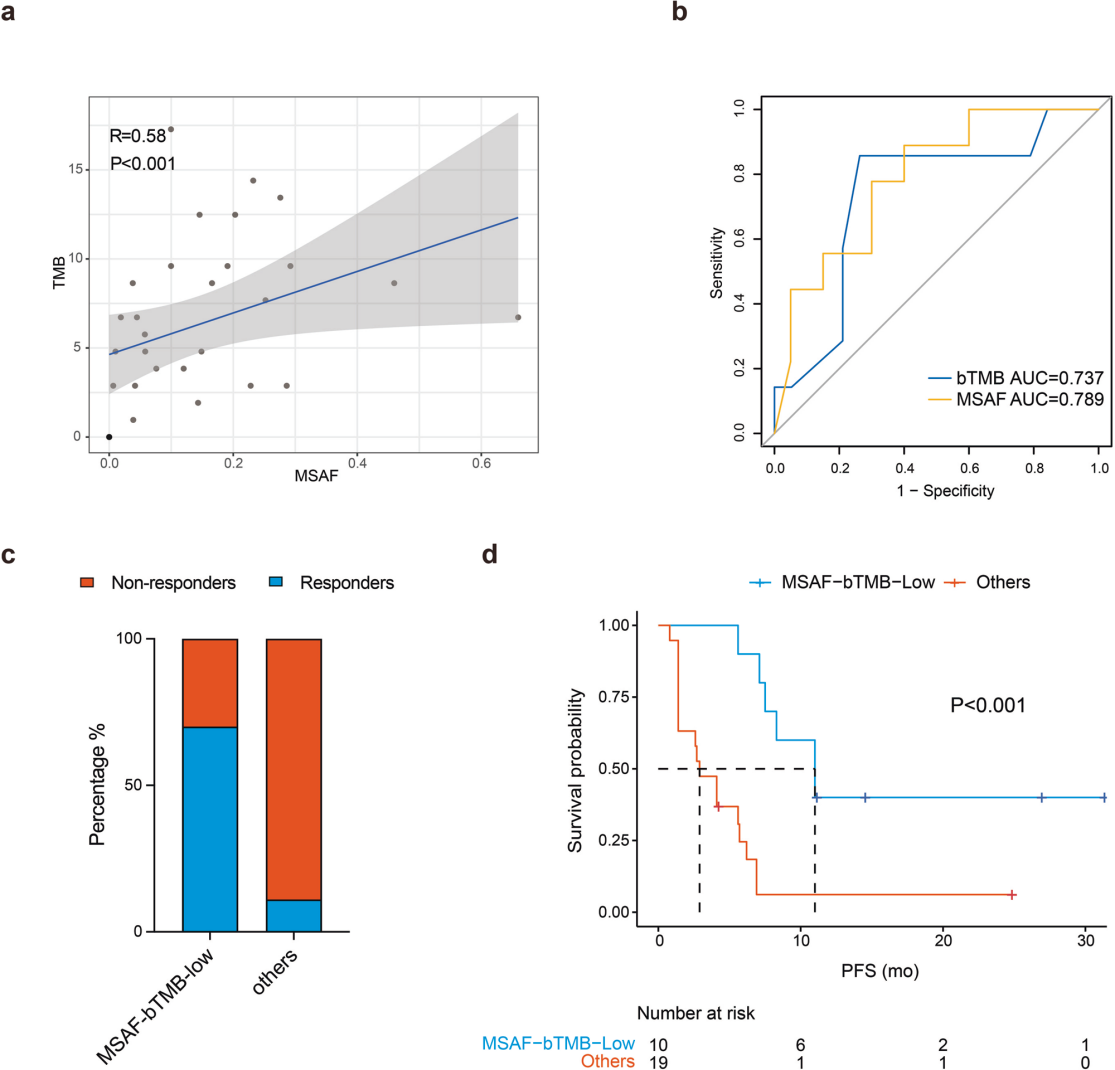
**a** Spearman analysis of correlation between MSAF and bTMB. **b** The area under the curve (AUC) of TMB and MSAF for treatment response (CR and PR). **c** Treatment response stratified by low bTMB and low MSAF *vs*. other bTMB and MSAF scenarios. **d** The Kaplan–Meier curves of PFS of efficacy-evaluable patients stratified by low MSAF and low TMB *vs*. other bTMB and MSAF scenarios

Spearman correlation analysis revealed significant positive correlation between the MSAF and bTMB (*r* = 0.58, *P* < 0.001) (Fig. 6a). MSAF had an AUC of 0.79 while bTMB had an AUC of 0.74 for objective response (Fig. 6b). Ten patients had both low bTMB and low MSAF while 19 patients had other scenarios of MSAF and bTMB. Seven patients with both low MSAF and low bTMB (70%, 7/10) showed objective response to anlotinib plus TQB2450 while only 2 patients (11%, 2/19) among those with other scenarios of MSAF and bTMB responded to the combination treatment (*P* < 0.001) (Fig. 6c). They also had a remarkably longer median PFS than those with other scenarios of MSAF and bTMB (11.0 *vs*. 2.9 months, *P* < 0.001) (Fig. 6d).

## Discussion

Pretreated aTNBC patients have rather limited therapeutic options and there is an urgent need for combination strategies to improve the outcomes of the patients and for effective biomarkers to guide treatment. However, lack of robust biomarkers predictive of response to immune therapy and antiangiogenic therapy hinders developing effective therapeutic strategies targeting biomarkers-selected patients. In the current report, we demonstrated that bTMB and MSAF could be potential biomarkers predictive of response to combined immunotherapy and antiangiogenic therapy in pretreated relapsed or metastatic TNBC patients. Notably, we found that patients low MSAF and low TMB had a remarkably greater objective response rate (70% *vs*. 11%) and remarkably longer PFS (11.0 months *vs*. 2.9 months) than patients with other scenarios of MSAF and TMB, suggesting a molecularly stratified approach for immune therapy and antiangiogenic therapy in patients with pretreated aTNBC. The identification of predictive biomarkers that enrich patients with TNBC who may derive PFS benefit is critical in the development and molecularly stratified treatment strategy for immune therapy and antiangiogenic therapy. Apart from bTMB and MSAF, the PI3K/AKT pathway was notably enriched in the altered genes, including *PIK3CA* and *PTEN*, which have been associate with resistance to immune therapy.^20,21^ To the best of our knowledge, this is the first investigation that establishes bTMB and MSAF as biomarkers associated with treatment response and survival outcomes of pretreated aTNBC patients receiving combined immunotherapy and antiangiogenic therapy.

TNBC could produce a myriad of neoantigens triggering a potent immune response and PD-L1 has emerged as a predictive marker for response to immune therapy. However, the utility of PD-LI has been hampered by inconsistencies across trials due to the use of different PD-L1 antibodies, staining of tumor cells *versus* immune cells, specific PD-L1 assays and different PD-L1 cutoffs, calling for the development of additional biomarkers predictive of response to immune therapy in TNBC.^22^ Among all breast cancer types, TNBC has the highest TMB and tissue-based TMB has been studied as a predictive biomarker for survival outcomes in TNBC.^23^ However, the clinical utility of tissue-based TMB is hampered by challenges in obtaining adequate amounts of tumor tissues for NGS and the invasive nature of the approach. Meanwhile, tumor-derived cell-free DNA in blood offers a ready and contemporaneous source of tumor DNA obtained *via* noninvasive liquid biopsy that allows determination of bTMB and mutation profiling of key tumor driver mutations; as a result, bTMB has emerged as a potential prognostic biomarker for immune therapy in TNBC.^24,25^ However, it remains to be explored whether bTMB could serve as a feasible surrogate for tissue-based TMB and be adopted in the clinic as an actionable predictive biomarker of combined immunotherapy and antiangiogenic therapy for aTNBC. In this report, we show that bTMB has an acceptable discriminatory power with an AUC of 0.74. Pathologic complete response to neoadjuvant chemotherapy with standard anthracycline- and taxane-based regimens has been demonstrated to be an effective prognostic indicator of long-term outcomes, especially in TNBC^3^. However, there was only limited overlap between features associated with pathologic complete response and event-free survival of clinical stage II to III TNBC patients receiving bevacizumab added to neoadjuvant chemotherapy.^26^ Our report shows that patients with low bTMB had a remarkably longer median PFS than those with high bTMB (7.3 *vs*. 4.1 months). The study findings suggest that bTMB could be predictive of treatment response and survival outcome of patients with pretreated aTNBC receiving combined immunotherapy and antiangiogenic therapy. Biomarker analysis of the KEYNOTE-086 study also showed an association between TMB and treatment response and survival outcomes in TNBC.^27^

TMB is not robust to sequencing depth as it is estimated by enumerating somatic mutations above a threshold frequency. When a substantial number of somatic mutations are present at low frequencies in a cancer sample, TMB may be overestimated, and the full mutant allele frequency spectrum should be included to achieve a robust estimation of TMB.^28^ MSAF is a valid bioinformatics tool for quantifying the tumor fraction of cell-free DNA in peripheral blood samples and has been investigated as a non-invasive predictive biomarker for immune therapy in lung cancer, but has not been evaluated in breast cancer.^29,30^ In this report, we show that MSAF has an AUC of 0.79 and could differentiate aTNBC patients who benefit from immunotherapy and antiangiogenic therapy from those who do not. At a cutoff of 10%, patients with low MSAF had a notably longer PFS (7.9 months *vs*. 2.7 months). Furthermore, patients who experience a posttreatment decline in MSAF had a significantly longer PFS than patients who saw an increase in MSAF (6.9 months *vs*. 2.0 months). We also found that MSAF positively correlated with bTMB. Remarkably, patients with both low MSAF and low bTMB had a significantly higher objective response rate those with other scenarios of MSAF and bTMB (70% *vs*. 11%) and a remarkably longer PFS as well (11.0 months *vs*. 2.9 months). Currently, no biomarkers have been established that predict treatment response to immunotherapy plus antiangiogenic therapy. Together with PD-L1 expression status, these biomarkers could guide stratification of aTNBC patients for the combination therapy, which could reduce toxicities and avoid the cost of unnecessary treatment. Our findings indicate that MSAF and bTMB could be potentially combined to guide the selection of pretreated aTNBC patients who derive from combined immunotherapy and antiangiogenic therapy.

Our study has several limitations. Our analysis is exploratory and was not prespecified in the phase Ib trial. The sample size in this study is relatively small; thus, the conclusions should be interpreted with caution. In addition, the primary endpoint of the phase Ib trial is the objective response rate; therefore, caution should be exercised when developing predictive biomarkers for survival outcomes. Another limitation of the study is the failure of inclusion of immune biomarkers including PD-L1 for predictive analysis. A comprehensive evaluation of genomic and immune activation biomarkers and angiogenesis biomarkers, and their combinatorial signatures may be required to identify biomarkers for survival benefit from combination immunotherapy and antiangiogenic therapy. Last, bTMB and MSAF were tested and validated in the same cohort and requires validation in an independent external cohort. The small sample size of the current study hinders direct application of bTMB and MSAF for testing their association with treatment response or using percentile as in the study by Samstein et al. ^31^ Currently, there is no consensus with regards to the cutoff for high TMB. A TMB ≥ 10 mut/Mb was used by the USA FDA for approving pembrolizumab for the treatment of high TMB TNBC.^32^ Liao et al. used the 75^th^ percentile for defining bTMB in advanced breast cancer patients receiving first line standard of care.^33^ Meanwhile, Samstein et al. used centile as well as quintile (top 20%) to define TMB-high patients.^33^ Meanwhile, no data is available on MSAF in breast cancer. A phase III trial (ClinialTrials.gov Identifier: NCT04405505) is being initiated, providing us the opportunity to examine the validity and robustness of bTMB and MSAF as biomarkers of treatment response to antiangiogenic therapy for breast cancer.

In summary, blood-based MSAF can effectively identify patients with pretreated aTNBC who could derive PFS benefit from immune therapy with novel PD-L1 inhibitor TQB2450 in combination with angiogenic inhibitor anlotinib, facilitating the development of molecularly stratified strategy for combined immune therapy and antiangiogenic therapy. The findings support further study of MSAF and the combined bTMB-MSAF classification as predictive biomarkers of treatment response in aTNBC patients.

## Materials And methods

### Patients and treatments

Patients with histologically or cytologically confirmed relapsed or metastatic TNBC received TQB2450 1200 mg (Chia Tai Tianqing Pharmaceutical Group) plus escalating doses of anlotinib (8, 10, and 12 mg). Tumor response was assessed per the Response Evaluation Criteria in Solid Tumors (RECIST), version 1.1 by investigators and by iRECIST in patients who developed PD. TNBC is defined by negative expression of estrogen receptor, progesterone receptor, and human epidermal growth factor receptor 2. Relapse is defined as disease progression despite receipt of prior anthracyclines and/or taxanes in the first line setting, or disease relapse or progression during or within 6 months of adjuvant therapy or neoadjuvant therapy. Objective response includes radiologically confirmed CR and PR, and disease control is defined by the attainment of CR, PR, or SD as the best overall response. PFS is defined as time from the start date of treatment to the date of the first documented disease progression or death of any cause. OS is defined as time from the start date of treatment to the date of death of any cause.

The trial was approved by the Ethics Committee of Cancer Hospital, Chinese Academy of Medical Sciences. All patients provided written informed consent prior to any trial activities.

### Targeted next generation sequencing (NGS)

Peripheral blood samples were collected prior to the start of treatment and at the time of disease progression. Circulating-free DNA was isolated using QIAamp Circulating Nucleic Acid Kit (Qiagen) per the manufacturer’s instructions. Each DNA sample based on Qubit quantification was fragmented and sheared DNA, approximately 170-bp in length, was used for end-repair, A-tailing, and targeted adapter ligation with unique identifiers, followed by amplification by polymerase chain reaction. Thereafter, all libraries were hybridized to an in-house panel of probes for 1021 genes. DNA sequencing was performed using the Gene+Seq-2000 sequencing system (GenePlus, Suzhou, China) per the manufacturer’s guidelines.

### Mutation calling

Terminal adaptor sequences and low-quality reads were removed separately from the raw data of paired samples using NCrealSeq (version 1.2.0, in-house) and NCfilter (version 2.0.0, in-house). Clean reads were aligned to the reference human genome (GRCh37) using Burrows-Wheeler Aligner (BWA, version 0.7.15-r1140). Duplicate reads were marked using realSeq and normal samples were marked using Picard tools (version 2.6.0). Single nucleotide variants (SNVs) and indels were detected using TNSCOPE (version 201808) and realDcaller (version 1.7.1), a software developed in-house to review hotspot variants, and the results of these analyses were merged using NChot (version 2.7.2, in-house) and then annotated to multiple public databases using NCanno (version 1.1.3, in-house). Copy number variations (CNVs) were called by CNVKIT (version 0.9.2). An in-house algorithm NCSV (0.2.3) was used to identify split-read and discordant read-pair to identify SVs.

The bTMB is calculated as the number of competent mutations divided by the length of the panel-covered genomic region (1.44 Mb). The blood-based MSAF is the highest allele fraction for confirmed somatic base substitutions regardless of their driver status. Three criteria were applied for competent mutations: (1) somatic but not germline mutation; (2) mutation located in the coding region, nonsynonymous SNVs/indels, including ±2 splices; (3) a mutation allele frequency ≥0.5%.

### GO and KEGG analysis

Gene Ontology (GO) annotation analysis and Kyoto Encyclopedia of Genes and Genomes (KEGG) pathway analysis were performed using the DAVID (Database for Annotation, Visualization, and Integrated Discovery) tool (https://david.ncifcrf.gov/).

### Statistical considerations

We estimated PFS and evaluated the association between bTMB, MSAF and PFS using the Kaplan-Meier method. Multivariate Cox regression analysis was used to adjust potential confounding factors. Kaplan–Meier curves were plotted and, together with log-rank tests, performed to calculate the hazard ratios (HRs). Spearman rank correlation analysis was used to examine the correlation between bTMB, MSAF and PFS. The proportional compositions of two or more variables were compared using Fisher’s exact tests. Median and mean values were compared using Wilcoxon tests. All statistical analyses were performed using GraphPad Prism (version 9.0), and R (4.2.0). For all analyses, *P* < 0.05 was considered statistically significant.

### Supplementary information

Supplemental Materials

## Data Availability

The data underlying this article are available in the article and in its online Supplementary Material.
