# Supplementary material for: Predictive biomarkers of response and survival following immunotherapy with a PD-L1 inhibitor benmelstobart (TQB2450) and antiangiogenic therapy with a VEGFR inhibitor anlotinib for pretreated advanced triple negative breast cancer
Source: Signal Transduct Target Ther. 2023 Nov 17;8:429. doi: 10.1038/s41392-023-01672-5 (PMC10654734; doi:10.1038/s41392-023-01672-5)

Supplementary Materials for

**Predictive biomarkers of response and survival following Immunotherapy with PD-L1 Inhibitor TQB2450 and antiangiogenic therapy with a VEGFR inhibitor Anlotinib for pretreated advanced triple negative breast cancer**

Yiqun Han, Jiayu Wang, Tao Sun, Quchang Ouyang, Jianwen Li, Jie Yuan, Binghe Xu

Correspondence to: xubingheBM@163.com

**This PDF file includes:**

Tables S1 to S2

Figures. S1

Table S1.

Clinicopathological characteristics of enrolled patients based on blood-based tumor mutational burden (bTMB).

| **Characteristics** | **The TMB cohort (N=26)** | | ***P* value** |
| --- | --- | --- | --- |
|  | **bTMB-high** | **bTMB-low** |  |
| No. of patients | 15 | 11 |  |
| Age, years |  |  | 0.296 |
| Median (range) | 51 (32-69) | 48 (42-66) |  |
| <65 | 14 | 10 |  |
| >=65 | 1 | 1 |  |
| ECOG PS |  |  | 0.115 |
| 0 | 12 | 11 |  |
| 1 | 3 | 0 |  |
| No. (%) of sites of metastatic disease |  |  | 0.312 |
| 1 | 1 | 3 |  |
| 2 | 6 | 4 |  |
| ≥3 | 8 | 4 |  |
| Site of metastatic disease |  |  |  |
| Non-visceral | 13 | 10 | 0.205 |
| Visceral | 9 | 4 | 0.399 |
| Liver | 4 | 1 |  |
| Bone | 7 | 2 |  |
| Lung | 5 | 3 |  |
| Lymph node only | 3 | 3 | 0.612 |
| Chest wall involvement | 4 | 4 | 0.597 |
| Neoadjuvant or adjuvant therapy |  |  |  |
| Anthracycline | 13 | 11 | 0.207 |
| Taxanes or anthracycline | 14 | 11 | 0.382 |
| Platinum | 8 | 6 | 0.951 |
| Number of previous systemic therapies |  |  |  |
| 1 | 12 | 8 |  |
| ≥2 | 3 | 3 |  |
| PD-L1 expression (CPS), No. (%) |  |  | 0.261 |
| <1 | 7 | 2 |  |
| ≥1 | 3 | 2 |  |
| Unknown | 5 | 7 |  |
| MSAF |  |  | 0.234 |
| High ≥13% | 9 | 4 |  |
| Low <13% | 6 | 7 |  |

Table S2.

Clinicopathological characteristics of enrolled patients based on blood-based maximum somatic allele frequency (MSAF).

| **Characteristics** | **The MSAF cohort (N=29)** | | ***P* value** |
| --- | --- | --- | --- |
|  | **MSAF-high** | **MSAF-low** |  |
| No. of patients | 13 | 16 |  |
| Age, years |  |  | 0.334 |
| Median (range) | 51 (32-62) | 48 (35-69) |  |
| <65 | 13 | 14 |  |
| >=65 | 0 | 2 |  |
| ECOG PS |  |  | 0.078 |
| 0 | 10 | 16 |  |
| 1 | 3 | 0 |  |
| No. (%) of sites of metastatic disease |  |  | 0.412 |
| 1 | 1 | 4 |  |
| 2 | 5 | 6 |  |
| ≥3 | 7 | 6 |  |
| Site of metastatic disease |  |  |  |
| Non-visceral | 12 | 14 | 0.808 |
| Visceral | 9 | 6 | 0.108 |
| Liver | 4 | 1 |  |
| Bone | 5 | 5 |  |
| Lung | 4 | 6 |  |
| Lymph node only | 2 | 4 | 0.321 |
| Chest wall involvement | 3 | 5 | 0.624 |
| Neoadjuvant or adjuvant therapy |  |  |  |
| Anthracycline | 12 | 15 | 0.879 |
| Taxanes or anthracycline | 12 | 14 | 0.672 |
| Platinum | 6 | 9 | 0.588 |
| Number of previous systemic therapies |  |  | 0.437 |
| 1 | 10 | 12 |  |
| ≥2 | 3 | 4 |  |
| PD-L1 expression (CPS), No. (%) |  |  | 0.498 |
| <1 | 4 | 8 |  |
| ≥1 | 2 | 4 |  |
| Unknown | 7 | 4 |  |
| TMB status, No. (%) |  |  | 0.125 |
| High (≥5 mutations/Mb) | 9 | 6 |  |
| Low (<5 mutations/Mb) | 4 | 7 |  |
| No somatic mutation | 0 | 3 |  |

Figure S1.

Gene mutations and treatment response in relapsed or metastatic triple negative breast cancer. Treatment response of efficacy-evaluable patients with mutated *versus* wildtype *TP53* (a), *MLL3* (b), *PIK3CA* (c), and *DNMT3A* (d). The Kaplan-Meier curves of progression-free survival (PFS) of efficacy-evaluable patients stratified by the alteration status of *TP53* (a), *MLL3* (b), *PIK3CA* (c), and *DNMT3A* (d).


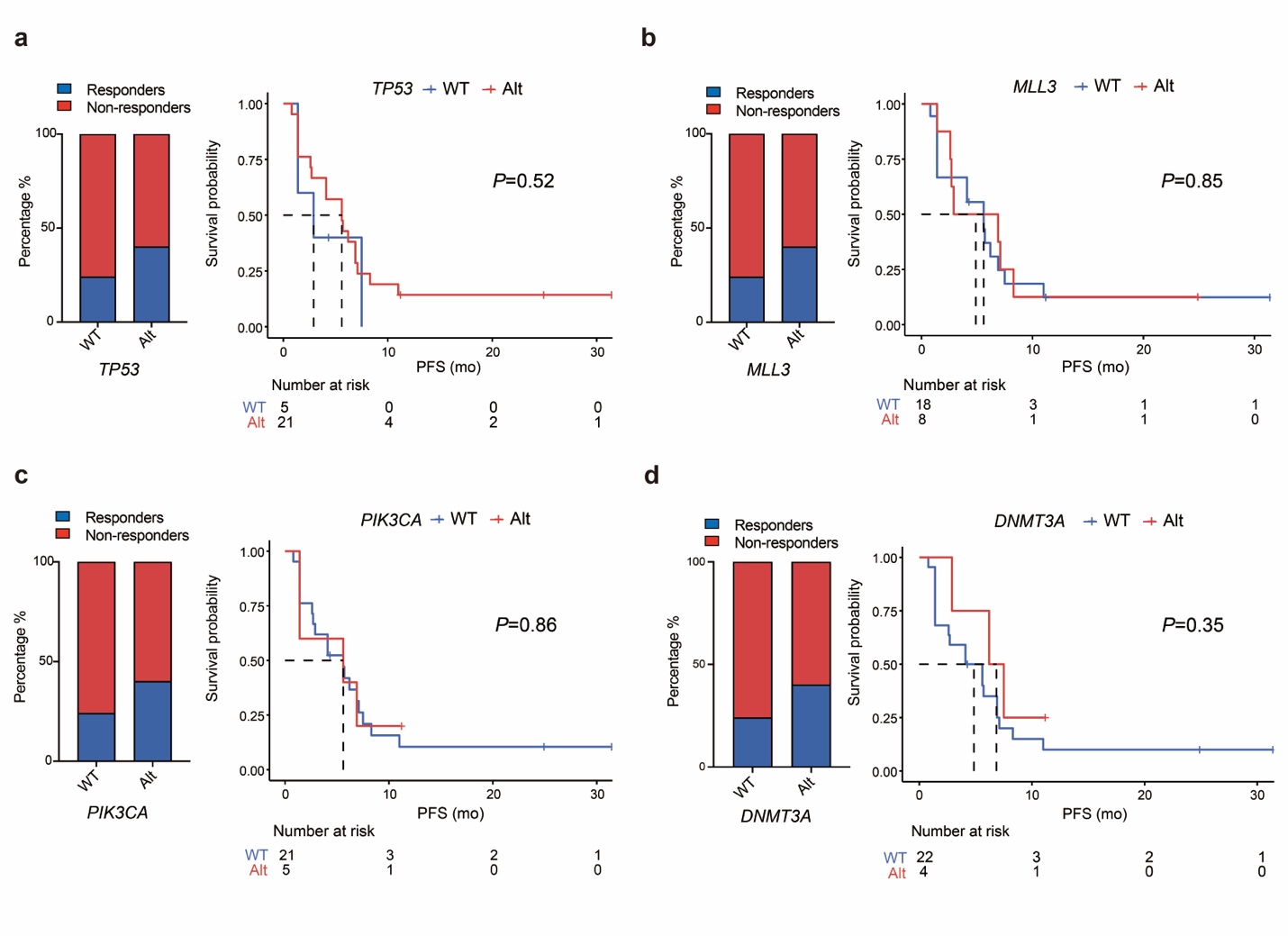

Supplement: Supplementary file 1 — Supplemental Materials [file 41392_2023_1672_MOESM1_ESM.docx]
